# Landscape transcriptomics as a tool for addressing global change effects across diverse species

**DOI:** 10.1111/1755-0998.13796

**Published:** 2023-04-11

**Authors:** Jason Keagy, Chloe P. Drummond, Kadeem J. Gilbert, Christina M. Grozinger, Jill Hamilton, Heather M. Hines, Jesse Lasky, Cheryl A. Logan, Ruairidh Sawers, Tyler Wagner

**Affiliations:** ^1^ Department of Ecosystem Science and Management The Pennsylvania State University University Park Pennsylvania USA; ^2^ Department of Biological Science Mount Holyoke College Hadley Massachusetts USA; ^3^ Department of Entomology The Pennsylvania State University University Park Pennsylvania USA; ^4^ W.K. Kellogg Biological Station, Department of Plant Biology, and Program in Ecology, Evolution, and Behavior Michigan State University Hickory Corners Michigan USA; ^5^ Huck Institutes of the Life Sciences The Pennsylvania State University University Park Pennsylvania USA; ^6^ Department of Biology The Pennsylvania State University University Park Pennsylvania USA; ^7^ Department of Marine Science California State University Monterey Bay Seaside California USA; ^8^ Department of Plant Science The Pennsylvania State University University Park Pennsylvania USA; ^9^ U.S. Geological Survey, Pennsylvania Cooperative Fish and Wildlife Research Unit The Pennsylvania State University University Park Pennsylvania USA

**Keywords:** conservation, gene expression, global change, landscape transcriptomics, wild populations

## Abstract

Landscape transcriptomics is an emerging field studying how genome‐wide expression patterns reflect dynamic landscape‐scale environmental drivers, including habitat, weather, climate, and contaminants, and the subsequent effects on organismal function. This field is benefitting from advancing and increasingly accessible molecular technologies, which in turn are allowing the necessary characterization of transcriptomes from wild individuals distributed across natural landscapes. This research is especially important given the rapid pace of anthropogenic environmental change and potential impacts that span levels of biological organization. We discuss three major themes in landscape transcriptomic research: connecting transcriptome variation across landscapes to environmental variation, generating and testing hypotheses about the mechanisms and evolution of transcriptomic responses to the environment, and applying this knowledge to species conservation and management. We discuss challenges associated with this approach and suggest potential solutions. We conclude that landscape transcriptomics has great promise for addressing fundamental questions in organismal biology, ecology, and evolution, while providing tools needed for conservation and management of species.

## 1 INTRODUCTION

In recent decades, advances in computation, statistics, remote sensing, and high‐throughput molecular biology have led to the development of new fields of ecological research that seek to link fine‐scale mechanisms with large scale processes and patterns (e.g., Buckley et al., 2010; Fisher et al., 2018; Heffernan et al., 2014; Lasky et al., 2020). In particular, landscape genomics has enabled ecological study of demographic history (Rougemont & Bernatchez, 2018), gene flow (Grummer et al., 2019), genetic drift (Toczydlowski & Waller, 2019), and local adaptation (Capblancq et al., 2022; Joost et al., 2007; Rellstab et al., 2015) across large spatial scales, with important insights for species conservation (Beer et al., 2022; Di Santo et al., 2022; Forester et al., 2021; Hohenlohe et al., 2021; Shaffer et al., 2022). We use “landscape” to mean terrestrial landscapes as well as aquatic ones (discussed more below). Here, we propose that a new field of “landscape transcriptomics” is emerging that can reveal the links between genetic variation, phenotypic variation, and landscape‐scale processes. The overall goal of landscape transcriptomics is to determine how patterns of gene expression across the genome link environmental variation across the landscape to organismal function and genetic differentiation among populations. Landscape transcriptomics integrates concepts from the fields of landscape ecology, macrosystems biology, landscape genomics, ecophysiology, and comparative population and ecological transcriptomics.

**FIGURE 1:**
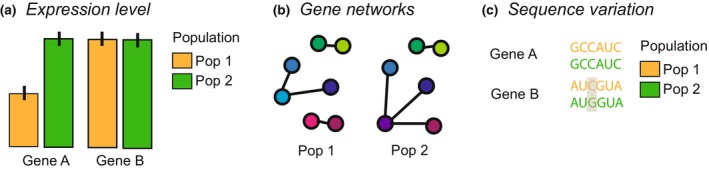
Information contained in transcriptome data collected from multiple populations across a landscape. (a) Gene expression levels may (or may not) vary between populations, (b) gene networks (inferred from correlations between different genes in their expression) may vary between populations, and/or (c) the sequence itself may vary between populations (Lopez‐Maestre et al., 2016; Piskol et al., 2013). Note that there could be biases in SNPs identified through transcriptome data compared to assumed “noncoding” DNA.

**FIGURE 2:**
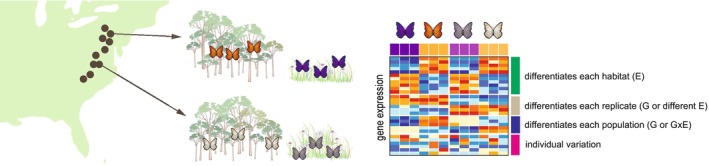
*Inferring patterns from landscape scale transcriptomic data.* The landscape transcriptomic approach involves sampling multiple locations. In this hypothetical example, butterflies are sampled from paired forested and meadow sites across a latitudinal transect in the eastern US. For simplicity, we then show hypothetical data for two of these paired sites (replicates), although additional replication would add power and make it easier to determine genes that are consistently associated with habitat differences (environmental main effect, E). Specifically, we show hypothetical gene expression data for three individuals from each population (note in practice, thousands of transcripts would be present in each transcriptome). Using a statistical model in which gene expression is predicted by environment (habitat comparison), replicate (the paired sites indicated by brown circles), and the interaction between the two (with population identity as a random effect or otherwise blocked), we would expect certain categories of genes to be present: (1) genes that are differentially expressed by habitat, (2) genes that are differentially expressed by replicate, (3) genes that are differentially expressed by population, and (4) genes that do not fall into these categories.

**FIGURE 3:**
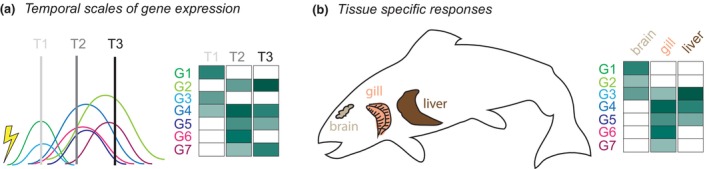
Challenges with transcriptomic data. (a) *Timing.* Gene expression has a time course that can vary by gene. In fact, expression of some genes may affect expression of other genes in a stepwise fashion – providing the mechanistic basis for gene networks. Therefore, when sampling occurs relative to an environmental stressor (yellow lightning bolt) can affect the patterns found. Gene expression levels are represented as a heatmap. (b) *Tissue specificity.* Different tissues will express different sets of genes and at different levels due to metabolic, physiologic, and internal environmental differences between them. Hypothetical gene expression data from three commonly assessed tissues in fishes are shown represented as a heatmap.

Several reviews on landscape genomics have, in passing, referred to the potential for other landscape ‐omics approaches that go beyond a focus on DNA sequence variation, including using transcriptomics, proteomics, and metabolomics (Balkenhol et al., 2017; Forester et al., 2021; Storfer et al., 2015). Although each of these landscape ‐omics approaches could advance our understanding of population responses to landscape‐level processes, in this paper, we argue that transcriptomics provides us with a wealth of unique information to do this (Figures 1 and 2), while presenting some distinct challenges (Figure 3). Moreover, techniques for transcriptomics have been developed to be high‐throughput, standardized, and readily applied to a diversity of organisms, which makes this approach more accessible than some other ‐omics approaches.

Transcriptomics is the study of the transcriptome – or the collection of RNA transcripts present in a given tissue of an organism at the time the sample is collected. For landscape transcriptomics, there are several classes of RNA molecules of potential interest that could be targeted with different techniques. These include messenger RNA (mRNA) that are typically transcribed into proteins, and various small RNAs (miRNA, piRNA, siRNA, etc.; Chen & Rechavi, 2022), often involved in gene regulation. Contemporary transcriptomic approaches use high‐throughput sequencing technology to obtain sequence data that is converted to quantitative gene expression data (Conesa et al., 2016; Todd et al., 2016; Wang et al., 2009); as such, there is a readout of how the genome reacted in the evolutionary past (via differences in sequence reflecting response to past selection) as well as the organismal past and present (via differences in identity and magnitude of gene expression reflecting prior experience and current conditions). Gene expression is the first phenotype, the precursor to all other phenotypic variation, and thus transcriptomes provide a unique opportunity to establish links between functional genetic variation and environmental heterogeneity in time and/or space, and their associated phenotypic response.

The field of landscape transcriptomics is now poised to transform how we approach ecology, evolution, and conservation for three reasons.

*First, the opportunity is now*. Two decades ago, Manel et al. (2003) laid the foundations of landscape genetics, which has evolved into landscape genomics (Balkenhol et al., 2017; Bragg et al., 2015; Grummer et al., 2019; Joost et al., 2007; Manel & Holderegger, 2013), integrating the use of genetic markers with landscape‐level processes. Since then, molecular tools and sequencing technologies have advanced at amazing speed allowing interrogation of sequence variation across the entire genome. These advances have enabled researchers to uncover the molecular mechanisms underlying ecological and evolutionary responses within both model and non‐model organisms (e.g., Ferrero‐Serrano & Assmann, 2019; Pfeifer et al., 2018; Sork et al., 2013). Leveraging transcriptomes provides the opportunity to integrate functional mechanisms with landscape‐level processes. The cost of RNAseq library prep and sequencing have decreased to <$200 a sample, and 3′ sequencing approaches (Lohman et al., 2016; Ma et al., 2019; Meyer et al., 2011) allow sample library preparation and sequencing for <$100 a sample, with prices continuing to fall. Thus, it is now financially feasible to conduct large‐scale sampling of transcriptomes (i.e., hundreds of individuals) across a landscape.

*Second, wild populations matter*. Sampling individual transcriptomes in the wild across environmental gradients is not only technically feasible, but also critical for linking responses occurring at the cellular level to population, community, and ecosystem‐level dynamics (Alvarez et al., 2015). The multitude of interacting stressors in nature (e.g., thermal, contaminants, biotic interactions, nutritional) suggests that sampling wild populations is fundamental to understanding how species respond to changing environments. Although sampling wild populations can make inferences of causal drivers difficult compared with controlled laboratory or mesocosm experiments, these experiments are often restricted to treatments that could miss important causal or interacting environmental factors. While the landscape transcriptomic approach explicitly involves collection of samples from across a landscape, in practice we suggest that complementing studies of transcriptomes from natural landscapes with controlled experiments (whether in the field, laboratory, or mesocosm) can provide a powerful approach for making inferences across multiple, often correlated, environmental axes.

*Third, time is of the essence*. Climate change and other anthropogenic disturbances are accelerating, impacting species' distributions, connectivity, and extinction risk (Barbarossa et al., 2021; Hughes, 2000; Hughes et al., 2018; Su et al., 2021; Telwala et al., 2013; Wagner et al., 2021; Walther et al., 2002). For some species of conservation concern, there may be few alternatives to sampling wild populations if we are to understand the evolution of adaptive phenotypes and their response to stressors. Fortunately, in many cases, transcriptomics can be performed using nonlethal sampling. Landscape transcriptomics thus provides an ability to establish mechanistic understanding of response to complex environments that may be used in predictive models for integration across scales of biodiversity (e.g., Bay et al., 2017). Information gained from such studies can be used to inform policy, conservation, and natural resource management decision‐making that is increasingly needed at a landscape‐scale.

## 2 FUNDAMENTAL QUESTIONS ADDRESSED BY LANDSCAPE TRANSCRIPTOMICS

Landscape transcriptomics is uniquely positioned to answer an array of questions important to ecologists, physiologists, evolutionary biologists, and conservation biologists. We emphasize the use of landscape‐scale observational transcriptome sampling with each of these questions but encourage complementary experimental approaches for validation and mechanistic interrogation. The questions are presented as discrete entities for clarity, but their integration is expected in practice.

1. How does expression across the genome (transcriptome) change across environmental gradients?
2. How do populations differ in their transcriptomic response to environmental gradients?
3. How can an understanding of the relationship between the environment and the transcriptome be used for management or conservation of target populations or species?

In addition to exploring how landscape transcriptomics can inform these questions, we provide a general workflow for a landscape transcriptomic study in Box 1 and explore statistical considerations for analysis of landscape transcriptomic data in Box 2.

BOX 1 **Considerations for designing and conducting a landscape transcriptomic study**

Collecting and analysing transcriptomes from across a landscape will not be trivial and careful decisions will need to be made each step of the way from study conception to study completion. Here, we highlight considerations for designing and conducting a landscape transcriptomic study. For basic information on how to conduct transcriptomics, the reader is encouraged to examine other reviews on that topic (Alvarez et al., 2015; Chung et al., 2021; Conesa et al., 2016; Lowe et al., 2017; Stark et al., 2019; Todd et al., 2016; Wolf, 2013).

1. ***Define study question(s) and priorities***, with special attention for the need for statistical power in the face of likely high variance in gene expression and potential trade‐offs between different study questions. The intended statistical analysis (point 5b) should be carefully considered now to inform sampling decisions (point 2).
2. ***Study design and field sampling*.** Replicated transects or paired site selection allow for more controlled variance than a random design, which may result in multiple intercorrelated environmental variables. Whether or not to pool samples is an important consideration and there is some disagreement on its utility (Alvarez et al., 2015; Rajkumar et al., 2015; Takele Assefa et al., 2020; Todd et al., 2016). Pooling increases the amount of RNA available, homogenizes within‐site variance, and is a cost‐effective strategy to increase population sampling and environmental gradient replicates. Pooling, however, will de‐emphasize genes with lower average expression and individual variation may be important for the study question or follow‐up questions (e.g., associating gene expression differences with sequence differences). Consider extraneous sources of variation, including temporal variation, temperature, humidity, or light intensity as well as biological variables like sex, reproductive status, or age class/developmental stage and either standardize, stratify, or record these variables as covariates. Consider nonlethal sampling approaches. Use a sampling method that ensures rapid stabilization of tissue samples to avoid transcriptional changes due to collection or handling stress.
3. ***RNA extraction*.** The ideal homogenization and extraction procedure will depend on the particular sample tissue and quantity. Because landscape transcriptomics will tend to involve many samples, adequate randomization of samples included in each extraction batch will be necessary to not confound technical error with other sources of variation.
4. ***Generate sequencing libraries and sequence*.** This is an area that is undergoing great technical improvement (Stark et al., 2019). An important consideration here is the particular sequencing technology to be used (e.g., short or long reads, single‐end or paired‐end, RNAseq or 3′seq) as this affects both library preparation and subsequent inference. The other important consideration is sequencing depth as greater depth enables more precise capture of genes with lower expression as well as more accurate gene expression inferences, but at a greater financial cost that likely sacrifices replicate numbers. The sequencing depth could be optimized after an initial pilot study by *in silico* subsampling to make saturation curves.
5. ***Bioinformatics***
  - *Counting transcripts*. There are many options to get from sequencing data to inferred counts of transcribed genes and other reviews have compared some of them (Chung et al., 2021; Conesa et al., 2016; Stark et al., 2019). Which tool to use depends in part on whether there is a reference transcriptome or genome available. Alternatively, a reference transcriptome could be assembled de novo from the data. Consider whether the reference represents population variation adequately so that read counts do not overreflect sequence divergence from the reference.
  - *Statistical analysis*. See Box 2 for a more in‐depth discussion of some statistical considerations for analysing counts of transcripts.
  - *Functional interpretation*. After genes have been identified in statistical analysis, characterizing their potential function using gene annotations and gene ontology terms is often desired to connect change in gene expression with a potential phenotypic effect. However, gene ontology databases are often underdeveloped for nonmodel systems (Courtier‐Orgogozo et al., 2020).
  - *Consider other ‐omics data*. Pairing transcriptomic data with whole genome sequencing data would allow interrogation of base‐pair changes linked to differences in transcription (eQTLs) or assessing signatures of past selection on implicated genes or putative regulatory regions for those genes. Similarly, transcriptomic data could be paired with information on chromatin accessibility (e.g., ATACseq) or methylation to understand environmental regulation of gene expression.
6. ***Conduct follow‐up experiments***. Identification of putative sources of variation in gene expression can lead to more controlled follow‐up designs such as greenhouse or mesocosm experiments for confirmation of hypothesized causal links between environmental variation and gene expression. Under these scenarios, manipulation of the expression of focal genes themselves using RNAi, viral‐mediated transfer of RNA, CRISPER/Cas, or related technologies could also be possible.

BOX 2 **Statistical considerations for landscape transcriptomics**

As described in the main text, landscape transcriptomic studies can potentially address several key questions. We illustrate with a toy example in Figure 2 how gene expression data collected across environmental gradients in a replicate manner can allow for description of consistent relationships between environmental variation and gene expression (Question 1) and generate hypotheses about population differences in gene expression response to the environment (Question 2), which will aid development of applied use of gene expression for conservation and management (Question 3). The data represented in Figure 2 can be statistically modelled as expression of a given gene being predicted by the fixed effects of the environmental variable of interest, the replicate of the environmental gradient, and the interaction between the two, with other fixed effects to incorporate covariates and random effects to incorporate other sources of variance (e.g., a genetic relatedness matrix, or other matrices capturing other forms of autocorrelation). This model will be more complex if a repeated measures design is implemented in which populations (or individuals) are sampled at more than one time point. As we describe below, tools developed for assessing differential gene expression are not necessarily adequate for analysing this model and we suggest several solutions in addition to calling for further tool development.

Transcriptomic data have some features which current tools for assessing differential gene expression are designed to deal with effectively. For example, transcriptomic data consist of many more genes (tens of thousands) than individual samples (tens to hundreds). Because gene expression data are based on counts of reads, the resulting data are non‐normal and the variance depends positively on the mean. These data are also potentially zero‐inflated. Commonly, expression of individual genes is modelled one‐by‐one, increasing the potential for Type 1 error, and so tools implement strategies for correcting for that issue. Experimental transcriptomic studies typically involved study designs with few factors (often one or two) with few levels and therefore relatively simple design matrices (which is why differential expression of genes is discussed, as a control is compared to an experimental group or one level of a treatment is compared to another). Current popular statistical packages for gene expression data (e.g., *DESeq2*: Love et al., 2014, *edgeR*: Robinson et al., 2010, *lima‐voom*: Law et al., 2014) have a similar workflow but differ in the details. In general, these tools first normalize the count data (i.e., adjust for differences between samples in sequencing depth; they might also account for gene length differences and other biases that occur with some sequencing libraries but not others), transform it to be appropriate for linear modelling through either a link function (gene expression data is similar to an overdispersed Poisson distribution, making negative binomial models and a logarithmic link function a common choice) or applying weights, and then apply a linear model. To deal with the typically small number of biological replicates, especially with respect to the number of genes quantified, analyses of gene expression estimate within‐gene variance using data for all or most genes (Todd et al., 2016).

One limitation of these current tools for analysing gene expression data is the inability to have mixed effects models with random effects. For example, although *lima‐voom* has a method for approximating a single random effects variable (the duplicateCorrelation function), *DESeq2* and *edgeR* do not have this capability, and none of these tools allow for more complex nested random‐effects designs or modelling of autocorrelation that likely occurs with landscape transcriptomic studies. For example, sequence information from the transcriptome itself or from ancillary genomic data would make it possible to build a matrix of similarity in genotype and thus control for relatedness by incorporating this matrix into the statistical model. Including relatedness in the model could lead to stronger inferences about genetic, environmental, and gene × environment interactive effects. Therefore, we encourage additional work on improving the statistical workflow. Until then, one could normalize counts from a bioinformatic pipeline using their favourite gene expression analysis package and then use those counts in the statistical modelling package of their choice whether that is a general linear mixed effects modelling package or a Bayesian modelling package, properly accounting for multiple testing. This will be computationally intensive and so access to a high‐performance computing cluster will be helpful.

Another feature of gene expression data is that the expression level of one gene is often correlated with the level of expression of other genes. One common approach to account for this and to explore the functional relevance of this coexpression is to cluster genes into groups based on correlated expression patterns. Summary statistics from these techniques (e.g., eigengene expression from *WGCNA*, Langfelder & Horvath, 2008) can then be used with any standard statistical modelling approach.

For landscape transcriptomics, a univariate gene‐by‐gene or module‐by‐module analysis is a good starting point. However, more flexible modelling approaches will likely be advantageous to accommodate the complex data structures that result from sampling organisms across space and time at broad spatial extents. Specifically, the use of multivariate statistical models that explicitly account for the dependency among genes and potential spatiotemporal or genetic dependencies will be advantageous. One such approach that may be useful for landscape transcriptomic data is generalized joint attribute modelling (*gjam*, Clark et al., 2017). Although motivated by the challenges in modelling species distributions, *gjam* can accommodate the data structures likely generated through landscape transcriptomic studies. Importantly, to borrow (and modify) language from Clark et al. (2017), *gjam* can accommodate the “big gene, small *n*” problem in transcriptomics, where the number of genes we are interested in modelling may exceed *n* by orders of magnitude. Certainly, there are other modelling approaches that could be applied to data generated by landscape transcriptomic studies, but the rigorous analysis of such data may require the application of new methods or the development of novel approaches.

### 2.1 How does expression across the genome (transcriptome) change across environmental gradients?

One goal of landscape ecology has been to understand how features of the environment influence the evolution and distribution of species (Turner, 1989). Landscape transcriptomics integrates the ecological and spatial contexts of landscape ecology with functional genomics and ecophysiology to provide a mechanistic understanding of factors underlying short‐ and long‐term responses to biotic and abiotic factors. Abiotic factors, including weather (encompassing temperature, precipitation, light), habitat structure (which can mediate the effects of weather, influence nutrient availability, etc.), and presence of contaminants (emerging and legacy chemicals) shape the distributions of populations based on their tolerance of these environmental factors. Biotic factors such as availability of food, predators, competitors, and disease are also often associated with landscape features. Thus, interactions between biotic factors, abiotic factors, and organismal gene expression patterns are expected to be complex. Identification of differentially expressed genes, functional groups, and/or coexpression networks offers great promise for providing deeper insight into the molecular mechanisms underlying physiological response across spatial contexts with application to higher orders of biodiversity across space and time (*sensu* e.g., Whitham et al., 2006).

There are examples of smaller‐scale transcriptomic studies that point to the potential of scaling up to larger landscape‐scale studies (i.e., the landscape transcriptomic approach). For example, a study in great tits, *Parus major*, found distinct differences between rural and urban birds in expression of >300 genes in liver and blood tissues, despite small sample sizes (6 rural males and 6 urban males, 1 site each, Watson et al., 2017). Specifically, urban birds upregulated genes involved in adaptive immune response, detoxification, and lipid metabolism. In the declining bumble bee, *Bombus terricola*, comparison of gene expression between foraging workers collected from agricultural sites (*n* = 18 workers, 6 sites) with those collected from nonagricultural sites (*n* = 12 workers, 4 sites) identified transcriptome differences consistent with exposure to pesticides and pathogens in agricultural sites (Tsvetkov et al., 2021). Thus, the transcriptomes of bees in agricultural landscapes suggested they were exposed more than bees in nonagricultural sites to fitness‐reducing agents (pesticides and pathogens), highlighting a tool for efficiently interrogating wild bee health (see also Box 3). A study of transcriptomes from the parasitic plant, *Striga hermonthica*, found unique expression patterns dependent on the biotic environmental factor of current host, where host varies across an agricultural landscape (maize = 3 sites or sorghum = 11 sites, 1 pool for each of two tissues from each site, Lopez et al., 2019). By increasing replicates of the environmental gradient of interest and choosing sites in a way that breaks correlations between the environmental variable of interest and other nuisance variables, the landscape transcriptomic approach could increase the inferential ability of these studies.

Where replicate environmental gradients are sampled, and a relationship between the focal environmental variation and gene expression is observed, this provides evidence that gene expression is dependent on the environmental factor of interest (e.g., habitat type in Figure 2). A number of critical questions relevant to finding such a statistical environment main effect can be answered, especially when individuals within a replicate are closely related (in the ideal case, clones), thus avoiding confounding genotype and environment. For example, what genes specifically have expression levels associated with the environmental variation of interest? Do these genes share similar functions? Does our knowledge of molecular physiology and systems biology allow us to infer how changes across the transcriptome work together to generate the physiological and whole‐organismal responses to the environment we observe? Are associations between gene expression and environmental variation seen across the whole transcriptome, suggesting a dramatic range of phenotypes respond, or are they narrower and modular, suggesting a specific subset of traits is responding? While assessing transcriptomes across a landscape offers a powerful avenue to addressing these questions, even more can be learned by incorporating additional information and utilizing more complex study designs as explained in the next section.

### 2.2 How do populations differ in their transcriptomic response to environmental gradients?

Above, we described how landscape transcriptomic studies, designed to evaluate populations across replicate gradients, can identify genes that are differentially expressed on average across replicates based on environmental variation (a statistical environment main effect, Figure 2). However, researchers are also interested in population differences in environmental response for various reasons (Des Marais et al., 2013; Saltz et al., 2018), including understanding how repeatable evolution is (Birkeland et al., 2022), what evolutionary processes maintain variation, and the role for plasticity in adaptive evolution (Anderson et al., 2012; Kingsolver & Buckley, 2017). In addition, these differences in response are relevant to understanding important applied questions such as the potential consequences of assisted migration (Chen et al., 2022) and the predicted differences between populations or species in response to habitat and/or climate change (Oomen & Hutchings, 2022).

In fact, we expect genes could be categorized into several classes based on whether their expression was correlated with the environment, the replicate, the population, or none of these (Figure 2). We expect one important area of future research will be to determine whether there are theoretical predictions for how the relative proportion of these different classes of genes might vary under different scenarios. Combining landscape transcriptomic data with landscape genomic data or the sequence data contained in the transcriptome (Figure 1) could allow characterization of the genetic relatedness between individuals and populations which could further aid interpretation of landscape transcriptomic data. As an extreme example, in Figure 2 if butterflies from a given geographic site were not genetically differentiated between habitats, but rather completely admixed, some of the genes that show population specific responses may actually be indicative of different genomic reaction norms, a genotype‐by‐environment (GxE) interaction. Landscape transcriptomics has the potential for establishing a hypothesis‐testing framework for the relationship between functional genetic variation and the environment that is essential for species conservation and management.

Populations may adapt to stressors either by changing mean expression (baseline expression) or transcriptional response (slope, transcriptional plasticity) to environmental fluctuations (Oomen & Hutchings, 2022; Rivera et al., 2021). Therefore, transcriptional plasticity itself may be a key trait under selection (Kenkel & Matz, 2017; Lasky et al., 2014; Logan & Cox, 2020), as it is a mechanistic basis for plasticity of other phenotypes. This flexible nature of transcriptomes creates novel challenges compared to DNA sequencing data (see more below). Careful sampling across time (Aubin‐Horth & Renn, 2009) could reveal such expression plasticity (e.g., sampling populations repeatedly across a heat wave). However, a substantial portion of transcriptomic response to environment may be maladaptive and indicate symptoms of a failure to maintain homeostasis, in which case additional information from experimental evolution (Ghalambor et al., 2015) or an understanding of the functional role of the differentially expressed genes is required.

Experiments could be used to test hypotheses generated from landscape transcriptomic studies. For example, one could determine whether apparent G × E has an alternative explanation such as genotypes within replicates segregating to particular environments – that is, a correlation between genotype and environment generating spurious genetic associations with environmentally‐responsive phenotypes (Saltz et al., 2018). Specifically, one could (1) bring populations into the laboratory for controlled comparisons (e.g., stress tests, Logan & Cox, 2020), or (2) conduct reciprocal transplant or common garden experiments (Gould et al., 2018; Kenkel & Matz, 2017; Oomen & Hutchings, 2022; Palumbi et al., 2014). Admittedly these types of experiments will be more logistically difficult than the correlational landscape transcriptomic approach described earlier, but they would allow more definitive parsing of G, E, and G × E effects.

Studies with the green anole, *Anolis carolinensis*, used a combination of a single latitudinal transect and laboratory stress tests to reveal mechanistic details of the evolution of thermal tolerance in ectotherms. Campbell‐Staton et al. (2017) collected anoles across a latitudinal transect in Texas both before and after a severe cold event (polar vortex), brought them into the laboratory, and tested gene expression in response to different acclimation temperatures. This study implicated temperature as the key abiotic factor driving adaptive differentiation across the latitudinal cline. In particular, coexpression modules that distinguished the populations across the latitudinal gradient showed shifts in expression in the southernmost population after the polar vortex that converged upon patterns more typical of northern populations (consistent with a phenotypic shift in cold tolerance). Further work with animals collected from this latitudinal transect revealed that cold tolerance is driven by physiological adaptations that enable more efficient oxygen consumption (Campbell‐Staton et al., 2018). Thus by pairing physiological measurements with tissue‐specific gene expression analyses, the authors were able to show that while temperature is a key stressor driving the evolution of *A. carolinensis* as populations move north (as previously established, Campbell‐Staton et al., 2016), more specifically it is respiration that was under selection. Recently, the nonmodel *A. cristellatus* was examined with a similar approach conducting experiments on anoles in the laboratory collected from four replicated pairs of forest versus urban populations (Campbell‐Staton et al., 2020), again showing that temperature is the key factor driving gene expression differences in populations across heterogeneous environments.

Finally, combining landscape transcriptomic data with landscape genomic data is a potentially powerful approach for understanding the evolution of gene expression. For example, it has been suggested that *cis* regulatory mutations are more likely to underlie adaptation than amino acid changes because of smaller effect size and a reduced chance of negative pleiotropy (Wray, 2007). Indeed, there are examples of local adaptation that use *cis* regulatory mutations (e.g., *EDA* in stickleback fish, O'Brown et al., 2015). However, there is often substantial gene flow across landscapes, which can swamp small‐effect, locally adaptive alleles. This suggests that only larger effect variants will be stably maintained (Yeaman & Whitlock, 2011). In this case, expression may alternatively evolve due to changes in regulators (*trans* mutation) such as transcription factors that control expression of many genes (e.g., *CBF2* in Arabidopsis, Des Marais et al., 2017; Monroe et al., 2016; Novillo et al., 2007). Such an integration of transcriptomic and genomic data across a single latitudinal gradient revealed rapid evolution of the relevant gene regulatory pathways in anoles (Campbell‐Staton et al., 2017).

### 2.3 How can an understanding of the relationship between the environment and the transcriptome be used for management or conservation of target populations or species?

Quantifying the impact of the potential stressors associated with complex environments is a fundamental aim of organismal biology, with implications for species management and conservation (Box 3). Environmental stressors include abiotic and biotic factors in natural and human‐mediated environments, such as exposure to pests or pathogens, extreme climatic events, pollutants, and nutrient limitations (Killen et al., 2013). The duration of exposure to environmental stressors need not be long to cause significant and long‐term effects to organismal health and function, and transcriptomes may provide an efficient evaluation of organismal response to the environment. For example, exposure to an environmental contaminant at one point in life, such as a pesticide, may trigger transcriptional changes that lead to developmental, physiological, or behavioural changes that impact the future health, longevity, and fitness of the organism. Yet while the pesticide itself may no longer be detected in the organism or environment when these changes are realized (Sponsler et al., 2019), the genetic networks underlying biological response may have been modified. Finally, exposure to stressors can influence gene expression in later generations after the stress has ended through a variety of mechanisms (Duempelmann et al., 2020; Liberman et al., 2019).

Landscape transcriptomics provides an efficient method to screen wild‐caught specimens' response to acute, chronic, or prior exposure to a stressor or combination of stressors. For example, glucocorticoids in vertebrate animals (involved in, among other things, the stress response) can directly affect gene expression because glucocorticoid receptors act as transcription factors for diverse gene networks (Weikum et al., 2017). If the transcriptional responses to particular stressors are well‐defined through prior work (including studies proposed in number 2 above), landscape transcriptomics can allow us to identify what stressors exist (or existed) in a particular location and potentially map these stressors across larger geographic regions (Connon et al., 2018; Jeffries et al., 2021; Semeniuk et al., 2022). Landscape transcriptomics potentially allows monitoring exposure to multiple and diverse stressors, simultaneously. Where tissue can be sampled in a nonlethal way (e.g., leaves in plants, gill tissue in fish, blood in multiple organisms), repeated measures of transcriptomes may provide an ability to differentiate the type of stress response (acute vs. chronic) that organisms exhibit. This approach might be logistically simpler than arrays of environmental monitoring systems and is increasingly biologically and ecologically relevant as transcriptional responses reflect immediate functional change associated with stress exposure. Thus, landscape transcriptomics identifies the environmental stressor, quantifies the response in the organism to the stress, and allows us to assess the long‐term consequences of exposure to the stress. In summary, transcriptomic signatures can be used to identify where to focus management and conservation strategies. We give specific examples of these opportunities in Box 3.

BOX 3 **Potential for landscape transcriptomics to inform conservation and management**

Landscape transcriptomics can be used to identify populations that are experiencing stress, allowing conservationists to target these sites to reduce levels of these stressors. Alternatively, by comparing populations that are experiencing a particular biotic or abiotic stressor, landscape transcriptomics can identify populations that are more resilient to this stress, which can be used to identify populations for assisted migration. Finally, by considering genome‐wide responses to a given stressor, landscape transcriptomics may reveal unexpected molecular and physiological pathways that respond to a given stressor, which can be leveraged to improve landscape conditions (such as availability of a particular nutrient) to mitigate the impacts of particular stressors. Below are examples in which these approaches have been or could be used in salmon, bees, corals, and plants.

**Salmonid management**

The development of transcriptional biomarkers, especially those that can be assessed nonlethally, have great promise for a variety of conservation and aquaculture applications. In fact, it has been argued that such nonlethal sampling for transcriptomics should be routinely used to study response to stressors in wild fishes, including thermal stress, salinity, disease, and contaminants (Connon et al., 2018; Jeffries et al., 2021; Semeniuk et al., 2022). For example, increasing water temperature represents one of the primary threats to cold water stenotherms such as salmonids and identifying thermal stress biomarkers is a conservation priority (Akbarzadeh et al., 2018). Houde et al. (2019) identified stressor‐specific gene expression biomarkers in Chinook salmon, *Oncorhynchus tshawytscha,* related to salinity and temperature in a multi‐stressor experimental design. Using the gill transcriptome, Jeffries et al. (2012) were experimentally able to identify physiological mechanisms involved in mortality and thermal stress in wild‐caught sockeye salmon, *O. nerka*. Such laboratory studies provide a strong scientific knowledge base for applying a landscape transcriptomic approach across multiple wild populations.

In addition, transcriptional response could identify adaptive genetic variants to be utilized in breeding or translocation efforts (Hayes & Banish, 2017). For example, brook trout, *Salvenlinus fontinalis*, have a narrow thermal window in which they can succeed (optimal temperature for growth = 12–16°C; upper critical maximum temperature = 28–31°C). One possible management approach would be to introduce genotypes from a donor population that are adapted to warmer streams northward as streams warm from climate change. However, identifying suitable populations to move is challenging as it is not always clear that just because a population is currently in a location it is locally adapted. There are often time lags in an environment shifting outside the range at which a population is optimally adapted and that population becoming extirpated. Thus, distinguishing between populations that are surviving vs. thriving is critical for translocation efforts.

**Bee conservation**

Bees are critical pollinators of flowering plants in both agricultural and natural landscapes (Ollerton et al., 2011). However, globally, populations of bees are showing significant population declines (Cameron et al., 2011; Soroye et al., 2020). Different factors have been attributed to bee declines, including pathogens, pesticides, poor nutrition due to reduced availability of flowering plants, and climate change (Wagner et al., 2021). Prioritizing conservation efforts is often difficult and based on incomplete information.

*A priori* knowledge of the pathways induced by specific environmental stressors from laboratory‐based experiments could be used to develop libraries of transcriptional stress responses which could be evaluated against observations in nature. In this way, these libraries of transcriptional responses may be used to infer landscape‐specific stressors in more complex environments. Such an approach would allow for ecologically defined functional categories of genes, rather than relying only on genes identified according to their annotated molecular function or known role in a model organism. Indeed, studies in honey bees demonstrated that transcriptional responses to pathogens and parasites included many more genes than canonical immune response genes (Richard et al., 2012), and similarly, responses to pesticides included many more genes than those involved in detoxification processes (Schmehl et al., 2014).

Sampling bees in the field and evaluating their transcriptome profiles can thus be used to identify populations that are experiencing specific stressors or combinations of stressors (Tsvetkov et al., 2021), which would allow conservationists to target those sites for additional management. Alternatively, if certain sites are known to have biotic and abiotic stressors, the transcriptomes of populations at these different sites could be evaluated to determine if some populations are more resilient to these stressors (i.e., not showing an elevated stress response in a stressful environment), and thus could be used for assisted migration. Finally, transcriptomic studies under controlled conditions or from field‐collected samples could determine how different biotic and abiotic factors may interact to reduce the impacts of a particular stressor, which could then inform management practices. For example, honey bees exposed to pesticides showed altered expression of genes related to metabolism and nutrition (Schmehl et al., 2014), which led to studies demonstrating that bees fed a pollen diet with a specific macronutrient ratio are more resilient to pesticide stress, and thus selecting species with pollen in this nutritional range for planting may improve fitness of bees in areas where they are consistently exposed to pesticide stress (Crone & Grozinger, 2021).

**Corals and climate change**

Climate warming has led to declines in coral populations globally, with coral reefs projected to disappear by 2030–2050 with no adaptation (Donner et al., 2005). As foundational species, like trees, coral loss could lead to the collapse of entire ecosystems. Reef restoration efforts and human interventions are currently underway to rebuild reefs decimated by hurricanes, disease, and marine heatwaves (National Academies of Sciences, Engineering, and Medicine, 2019). These efforts require the identification of stress resistant individuals that can withstand future conditions. Landscape transcriptomics is proving to be a useful technique for identifying heat tolerant coral individuals and populations. For example, baseline gene expression across locations has been used to identify coral gene modules predictive of future heat tolerance in American Samoa (Naugle et al., 2021) and/or recovery following bleaching (Thomas et al., 2018). Surveys of transcriptomic resilience (defined as return to baseline or control levels of gene expression, Franssen et al., 2011; Rivera et al., 2021) are also underway on field‐collected corals worldwide (e.g., Voolstra et al., 2021) to determine the generality and limitations of transcriptomic markers for conservation purposes across populations.

**Plant breeding**

Plant breeding has historically focused on selection for yield under optimum field conditions. In the face of climate change and the need to reduce reliance on agricultural inputs, breeding programs and the research community are giving greater attention to crop stability – the capacity to maintain productivity under changing or stressful environmental conditions. Transcriptomic approaches have been used extensively, typically in the greenhouse or growth chamber, to describe crop molecular responses to a broad range of stressors such as drought, extremes of temperature, salinity, nutrient deficiency, and pathogen attack (e.g., Secco et al., 2013), providing potential libraries of transcriptomic stress response. Transcriptome data from agricultural fields across large landscape scales could provide one more layer of information to help understand why certain varieties tolerate given stressors better than others, and, importantly, to predict which associated genotypes will show superior tolerance further on in a breeding programme. Where such studies are applied to traditional locally adapted varieties in their native environments, there are strong parallels with the goals of landscape transcriptomics as applied to wild populations (e.g., Hu et al., 2022).

## 3 CHALLENGES AND SOLUTIONS

### 3.1 Confounding variables in field sampling

While landscape transcriptomics can address a variety of questions, each with their own considerations, there are a number of general challenges facing collection, analysis, and interpretation of transcriptome‐level data based on samples from natural environments. Whether interested in documenting how transcriptomes vary with specific conditions, which environmental conditions most alter transcriptomes, identifying genes involved in adaptive phenotypes, or using transcriptional data as bioindicators, there is an overarching problem of environmental stochasticity. In addition, a random selection of individuals from the environment may be influenced by numerous, sometimes unknown, variables. A given organism's transcriptome can vary due to factors other than environmental stressors, and how individual variation in transcriptomes naturally change in response to environmental variation over short‐ and long‐time scales is poorly understood for most organisms. For example, circadian rhythms can have a large influence on transcriptional profiles (Bonnot et al., 2021; Prokkola & Nikinmaa, 2018, see below); in addition, developmental age (Li et al., 2016; Rahman et al., 2021), sex (Brivio et al., 2020; Hellmann et al., 2020), reproductive status (Niño et al., 2013), and immune/disease condition (Doublet et al., 2017) all can influence transcriptional patterns (Grozinger & Zayed, 2020). These factors can generate considerable variance in gene expression that can make it challenging to separate transcriptomic responses to the environment from that of other sources of individual variance.

One (partial) solution to this problem is to record extensive data on the potential confounding variables for inclusion in statistical models (e.g., weather conditions if weather is not the focus of the study) and to seek to control extraneous variables (e.g., time of day) when sampling. Statistically controlling for potential confounding variables will require an increase in sample size, increasing costs in time and money. The reason we believe these studies are feasible, however, is that the cost of sequencing continues to come down.

As discussed in number 2 above, populations may vary in their transcriptional response, and this can make detection of an average population response to the environment difficult. When possible, studies should attempt to have good replication of transects/pairs used in environmental gradient comparisons. Quantitative genetics has shown there are trade‐offs between increasing the number of additional families sampled versus sampling additional individuals within a family, and the resolution of these trade‐offs depends on the particular research question. For example, when estimating additive genetic variance, increasing the number of families rather than individuals often gives better “bang‐for‐buck” (Lynch & Walsh, 1998). Similar trade‐offs for landscape transcriptomic studies surely exist and their resolution will also depend on the research question. For example, if the question is about genes whose expression are most affected by a landscape variable, then sites (and replicates of environmental gradients) should likely be prioritized over individuals within sites. However, the best approach is likely the opposite if the interest is primarily in gene expression variation within sites. Simulation studies could help define these trade‐offs (Wagner et al., 2016). Finally, pooling samples is common, especially for small organisms or tissues, and these will amplify mean differences between populations and downplay individual variation. In summary, how best to sample for landscape transcriptomic studies is an open and not trivial question that will depend to a large extent on study question and species of interest. While such challenges have been covered elsewhere (e.g., Alvarez et al., 2015), we summarize some specific issues and considerations related to landscape transcriptomics below.

### 3.2 Temporal scales of gene expression

To match temporal changes in the environment, most organisms have evolved biological rhythms in behaviour, physiological processes, and regulation of gene expression (Figure 3a). Circadian rhythms in gene expression are broadly found across taxa, from prokaryotes to plants, fungi and animals, and may be particularly important for sessile species. For example, approximately one third of expressed *Arabidopsis* genes are known to be circadian regulated (Covington et al., 2008). Further, circadian rhythms can interact with other temporal rhythms in gene expression. Intertidal mussels are known to oscillate the expression of >40% of their genes in response to circadian and tidal rhythms (Connor & Gracey, 2011). Interestingly, the majority of oscillatory genes (80%–90%) are tied to circadian rhythms rather than tidal rhythms that coincide with extreme exposure to temperature and desiccation stress. Reef‐building corals are also known to modulate gene expression (thousands of genes) in response to seasonal temperature, the lunar cycle, and diurnal rhythms (Wuitchik et al., 2019). These studies highlight the importance of designing experiments that include multiple timepoints and account for temporal environmental changes that may not be aligned across geographic regions (e.g., the timing of tides, phenology). Alternatively, a more cost‐effective strategy would be to choose a single point in time and development (and tissue, see below) and restrict inference to that particular moment (e.g., morning temperature effects on gene expression in adult nonreproductive fish gills, or daylength effects on gene expression at midday in 1‐month old plant leaves).

### 3.3 Tissue specific responses

Another important consideration for landscape transcriptomic studies involving multicellular organisms is tissue choice. For early life stages or smaller organisms (e.g., arthropods, fish fry, seeds), transcriptomic studies have often homogenized the whole organism or even pooled across individuals for practical reasons. However, tissue‐specific differences in gene expression (Figure 3b) can be substantial and may differ across populations. For example, in a study of Atlantic killifish, *Fundulus heteroclitus*, 76% of metabolic genes were differentially expressed among brain, heart, and liver tissues (Whitehead & Crawford, 2005). Of these, only 31% of tissue‐specific differences were consistent in expression among fish originating from three populations along the U.S. east coast. Even within a single organ, differences between cell types of that tissue can be substantial (Colquitt et al., 2021; Seyfferth et al., 2021). Therefore, it is necessary to have careful consideration of which tissue is chosen and acknowledgement of the limits of inference created by that choice.

### 3.4 Defining spatial scale

A central feature at the heart of landscape ecology is the issue of scale. The term “landscape” is often understood to mean very large‐scale geographic areas (e.g., continental). However, landscapes can be defined in reference to spatial heterogeneity rather than physical geographic distance *per se* (Balkenhol et al., 2017), with the scale of landscapes dependent on organism body size and dispersal ability. For example, a large animal moving from one edge to another of a 1 km^2^ forest plot might not experience high environmental heterogeneity, so this area would not constitute a landscape. On the other hand, this same area could be a landscape for an insect, and even more so for a microbe. Small features like rocks and logs will alter conditions of the local microenvironment for small organisms (e.g., temperature, light, humidity), and thus could provide gradients conceptually comparable to macroscale latitudinal and elevational ones. Given the potential to use very small areas, microbial systems could serve as experimentally tractable models for probing landscape transcriptomic questions relevant to macroscopic organisms at larger scales, in addition to addressing questions relevant specifically to microbes.

### 3.5 The benefits of supplementing with a controlled design experiment

An advantage of landscape transcriptomics is that in natural environments, we may be more likely to detect genes that are only expressed in natural conditions. However, interpretation of results can be complicated by the multivariate nature of environments and lack of true replicates and controls. When possible, we advocate considering controlled, replicated experiments to be done either first or in combination with landscape transcriptomics to aid inference. This will be especially important when asking questions related to transcriptional plasticity.

## 4 CONCLUSION

What's in a name? It could be argued that because landscape transcriptomics is adjacent to other currently named fields, there is no need for a separate label. And it turns out that “landscape transcriptomics” was in fact coined >10 years ago (Hansen, 2010) and continues to be mentioned obliquely in some reviews of landscape genomics. However, we argue that there are unique strengths and challenges to using transcriptomics in a landscape context that call attention to important study design details and interpretation. By emphasizing the label “landscape transcriptomics” and describing the strengths and challenges of this approach, we hope to generate exciting new research effectively capitalizing on sampling transcriptomes from across the landscape. We also see the great potential for this approach to generate tools for conservation and management of species.

## AUTHOR CONTRIBUTIONS

Jason Keagy conceived of the idea for a review on this topic and organized the authors, including group discussions about landscape transcriptomics. Jason Keagy wrote the initial draft and all other authors contributed to writing text and editing. Jason Keagy drew all figures with input from all authors.

## CONFLICT OF INTEREST STATEMENT

The authors have no conflicts of interest to declare.

## Data Availability

Data sharing is not applicable to this article as no new data were created or analysed in this study.
